# A Compact Dual-Oblique-Fiber Heterodyne Phase-Shifting Point Diffraction Interferometer

**DOI:** 10.3390/s26113452

**Published:** 2026-05-29

**Authors:** Yongjie Wang, Conghui Zhu, Wenxi Zhang

**Affiliations:** 1 Aerospace Information Research Institute, Chinese Academy of Sciences, Beijing 100094, China; wangyongjie22@mails.ucas.ac.cn (Y.W.); zhuch@aircas.ac.cn (C.Z.); 2 School of Optoelectronics, University of Chinese Academy of Sciences, Beijing 100049, China

**Keywords:** heterodyne phase-shifting interferometry, point diffraction interferometer, oblique fiber, wavefront sensing, surface measurement, optical testing

## Abstract

Point diffraction interferometers (PDIs) utilize a near-ideal spherical wavefront generated by point diffraction as the reference, providing a high-quality measurement benchmark independent of reference surface quality. In this work, a compact dual-oblique-fiber heterodyne phase-shifting point diffraction interferometer (DOF-HPSPDI) is proposed. A dual-oblique-fiber point diffraction wavefront generator (DOF-PDWG) is designed to generate the reference and measurement beams separately. The proposed configuration enables efficient utilization of the divergence of the fiber-generated diffracted wavefront, while the reflective structure at the fiber end faces allows the two beams to propagate along a common path. In addition, the close spacing between the two oblique fibers minimizes system errors. Heterodyne phase-shifting interferometry (HPSI) is employed to retrieve the wavefront phase from the interferograms. Theoretical system errors are analyzed through simulations, and experiments verify the feasibility and stability of the proposed system. This work provides a low-cost, compact, and highly stable point diffraction interferometer, offering a promising device for high-precision optical testing and sub-aperture stitching of large-aperture optical components.

## 1. Introduction

With the continuous advancement of science and technology, high-precision optical components have been widely applied in high-end equipment manufacturing, the semiconductor industry, and astronomical optics [1,2,3,4]. The surface figure accuracy of these components has become a critical foundation for the performance of advanced optical systems. Therefore, high-precision surface metrology is of great importance.

Phase-shifting interferometry (PSI) is a non-contact, full-field, and high-precision measurement technique, and has become one of the most widely used and effective methods for optical surface metrology [5]. Conventional interferometers, such as the Twyman–Green and Fizeau interferometers, are essentially relative measurement systems [6]. In particular, the Twyman–Green interferometer suffers from non-common-path errors due to its configuration. Moreover, the measurement accuracy of these systems is fundamentally limited by the quality of the reference surface. To overcome these limitations, several absolute testing techniques have been developed, including the two-sphere method [7], rotation–translation method [8,9,10], and random ball method [11]. However, these approaches are generally complex and impose stringent requirements on mechanical alignment and motion accuracy.

Point diffraction interferometers (PDIs) generate a high-quality spherical wavefront through a micro-aperture and use it as a reference, thereby eliminating the dependence on reference surface quality [12]. According to the generation mechanism, PDIs can be categorized into pinhole-based and fiber-based configurations. Among them, fiber-based point diffraction techniques have attracted considerable attention due to their low cost, high energy efficiency, and long lifetime.

Sommargen et al. first applied fiber point diffraction to the measurement of extreme ultraviolet (EUV) optical components [13,14]. In their scheme, the diffracted wavefront is divided into reference and measurement beams, resulting in inefficient utilization of optical energy and a limited measurable aperture. In addition, a short-coherence light source is required to avoid phase shifting by moving the test surface. To address these issues, Kihm et al. proposed an oblique fiber-based point diffraction generator, which spatially separates the emitted wavefront from the reflected measurement wavefront, enabling full utilization of the diffracted wavefront [15,16]. In their scheme, the introduction of a beam splitter (BS) between the reference and measurement paths leads to non-common-path errors. Subsequently, Matsuura et al. proposed a dual-fiber point diffraction interferometer, in which two oppositely oriented fibers generate the reference and measurement beams, respectively [17,18,19]. However, due to incomplete overlap between the two beams, significant non-common-path errors are still introduced. To mitigate these errors, a lensless configuration and complex diffraction propagation algorithms were employed, which strongly depend on accurate system parameters.

In addition, most existing PDI systems employ mechanical phase shifting [20]. In contrast, heterodyne phase-shifting interferometry introduces a small frequency difference between two beams using an acousto-optic modulator (AOM), thereby generating a stable and continuous phase shift in the time domain, which has been demonstrated to provide superior phase stability [21,22,23,24,25].

Motivated by the above considerations, this paper proposes a dual-oblique-fiber heterodyne phase-shifting point diffraction interferometer (DOF-HPSPDI). A dual oblique fiber point diffraction wavefront generator (DOF-PDWG) is first designed. Owing to the oblique fiber configuration, the emitted wavefront and the reflected measurement wavefront can be spatially separated, enabling full utilization of the diffracted wavefront. Meanwhile, the end faces of the two fibers are positioned on the same plane and embedded within a common fused silica substrate, allowing the reference and measurement beams to propagate in a common path within the imaging system, thereby eliminating non-common-path errors. Furthermore, the distance between the two fibers is minimized to suppress errors caused by source separation.

On this basis, heterodyne phase-shifting interferometry is employed to achieve high-stability phase modulation via a small frequency difference between the two beams, enabling high-precision digital phase demodulation.

The deviation between the wavefront generated by the oblique fiber and an ideal spherical wavefront is theoretically analyzed. The system errors induced by the incomplete overlap of the two fibers are also investigated and compensated. Experimentally, the DOF-PDWG is fabricated, and a DOF-HPSPDI system is established. The feasibility and repeatability of the proposed method are verified through experiments.

Compared with previous schemes, the proposed structure first eliminates the wavefront error introduced by the BS. In addition, in our configuration, the spacing between the two fibers can be minimized to reduce system error, while allowing the measurement beam and the reference beam to propagate along the same optical path within the imaging system. Furthermore, heterodyne phase shifting is employed as the phase-shifting approach, which further improves the stability of the system.

The proposed structure features low cost, high stability, and a compact configuration while maintaining high measurement accuracy. Owing to its compactness, the proposed system also shows potential for specialized applications, such as in-situ sub-aperture stitching measurements of large-scale optical components [26,27].

## 2. Methods

### 2.1. Design and Working Principle of DOF-PDWG

**Figure 1:**
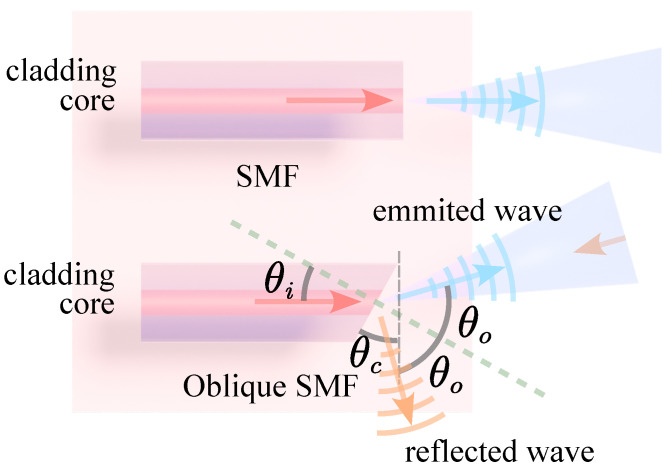
Illustration of an oblique fiber and its emission characteristics.

As shown in Figure 1, the principle of an oblique fiber is illustrated. For a conventional single-mode fiber (SMF), the emission direction is aligned with the propagation direction of light inside the fiber. When the fiber end is cleaved at an angle $\theta_{c}$, due to the refractive index difference between the fiber and air, the emitted beam is refracted at the oblique end face and exits at a certain angle. According to Snell’s law, the relationship can be expressed as

(1) $$n_{f}\sin\theta_{i}=\sin\theta_{o},$$

where $n_{f}$ is the refractive index of the fiber, $\theta_{i}$ is the incident angle with $\theta_{i}=\theta_{c}$, and $\theta_{o}$ is the output angle.

**Figure 2:**
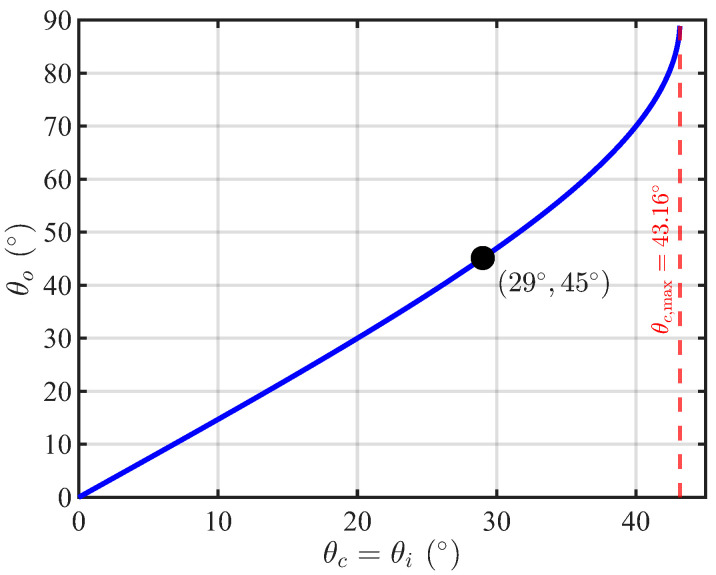
Relationship between the cleaving angle and the output angle.

Figure 2 shows the relationship between the incident angle and the output angle. Since the light propagates from an optically denser medium to an optically rarer medium, total internal reflection occurs when the incident angle reaches the critical angle $\theta_{c,max}$.

If the emitted beam is reflected back along its original path, the returning beam will be reflected again at the oblique fiber end face. The angle between the emitted beam and the reflected beam is $2\theta_{o}$. Therefore, the oblique fiber not only generates a point diffraction wavefront but also functions as a beam splitter (BS), separating the emitted and reflected beams. In this work, the oblique angle is chosen as $\theta_{c}=29^{\circ }$, corresponding to an output angle of $\theta_{o}=45^{\circ }$. As a result, the angle between the emitted and reflected beams is $2\theta_{o}=90^{\circ }$.

The cleaving angle of 29°, corresponding to a beam separation angle of 90°, was selected as a reasonable structural design after considering both sufficiently low point-diffraction wavefront error and adequate beam separation. In addition, this configuration helps avoid mechanical interference between the measurement path and the reference path. Furthermore, the selected design value is consistent with those reported in previous studies [16].

**Figure 3:**
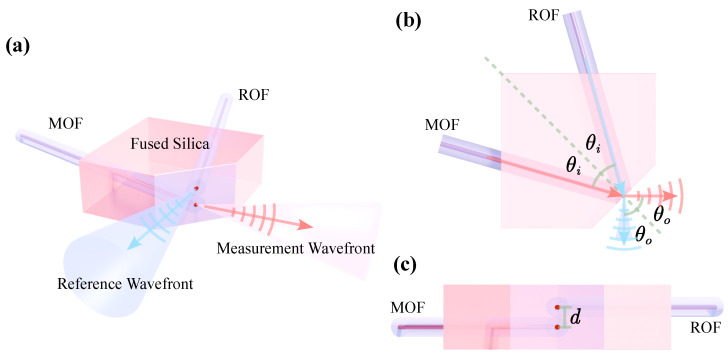
Schematic diagram of the dual-oblique-fiber point diffraction wavefront generator (DOF-PDWG). (**a**) Perspective view. (**b**) Top view. (**c**) Output-end view.

As shown in Figure 3, the structure and working principle of the dual-oblique-fiber point diffraction wavefront generator (DOF-PDWG) are illustrated. The overall perspective view, top view, and output-end view are presented in Figure 3a–c, respectively.

Two oblique fibers are embedded in a fused silica block, which is made of the same material as the fibers. Both fibers have the same oblique angle $\theta_{c}$ and are symmetrically arranged with respect to the normal of the output surface. Therefore, the angle between the two fibers is $2\theta_{c}$. Since both fibers share the same output angle $\theta_{o}$, the angle between their emitted beams is $2\theta_{o}$, which equals $90^{\circ }$ due to symmetry.

In practice, the two fibers cannot be perfectly overlapped. Instead, they are arranged in close proximity as shown in the figure. Given that the cladding diameter of the fiber is 2b=125 μm, the distance between the two emission points is d = 125 μm.

One oblique fiber is designated as the measurement oblique fiber (MOF), while the other serves as the reference oblique fiber (ROF). According to the working principle of the oblique fiber, the measurement beam emitted from the MOF, after being reflected back along the original path, will interfere with the reference beam emitted from the ROF.

Therefore, the proposed DOF-PDWG is capable of simultaneously providing a reference wavefront and a measurement wavefront, while also functioning as a beam splitter. The use of oblique fibers ensures efficient utilization of the wavefront divergence angle, and the compact structure minimizes the spacing between the MOF and ROF, thereby reducing system errors.

### 2.2. System Layout of the DOF-HPSPDI

**Figure 4:**
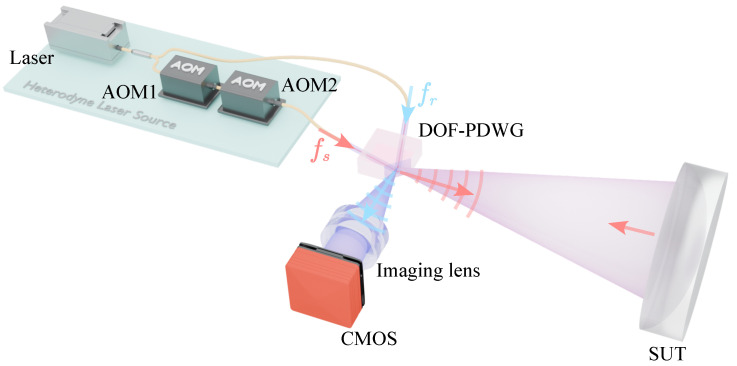
Schematic of the dual-oblique-fiber heterodyne phase-shifting point diffraction interferometer (DOF-HPSPDI) system configuration.

As shown in Figure 4, the system consists of a heterodyne light source, a DOF-PDWG, and an imaging module. In the heterodyne light source, a laser with a wavelength of λ=632.8nm is used. The emitted beam is split into two paths by a fiber coupler. One path is directly connected to the reference oblique fiber (ROF) of the DOF-PDWG and serves as the reference beam, while the other path is connected to the measurement oblique fiber (MOF) after passing through two cascaded acousto-optic modulators (AOM1 and AOM2), which are driven by radio-frequency (RF) signals to control the frequency shifts, serving as the measurement beam.

After frequency modulation by the AOMs, the measurement beam acquires a frequency shift Δf. Therefore, the relationship between the measurement beam frequency $f_{s}$ and the reference beam frequency $f_{r}$ can be expressed as

(2) $$f_{s}=f_{r}+\Delta f.$$

The wavefront phase of the beam reflected by the sample under test (SUT) contains the surface information of the SUT, which can be expressed as

(3) ϕ(x,y)=2kL(x,y),

where ϕ(x,y) is the wavefront phase, L(x,y) represents the surface profile of the SUT, and k=2π/λ is the wave number.

The measurement beam carrying the SUT surface information is reflected by the DOF-PDWG and interferes with the reference beam emitted from the ROF. Since the wavefront generated by the DOF-PDWG is a near-ideal spherical wave, the phase information contained in the interference pattern is solely introduced by the SUT, without contributions from a reference surface or other optical components. Hence, the proposed system is a point diffraction interferometric system.

By employing heterodyne phase-shifting interferometry, the phase distribution introduced by the SUT can be retrieved from the interference signal, and thus the surface profile of the SUT can be reconstructed. The heterodyne phase-shifting technique and phase retrieval method will be described in Section 2.3.

### 2.3. Phase Retrieval Based on HPSI

In heterodyne phase-shifting interferometry (HPSI), a small frequency offset Δf is introduced between two coherent beams, resulting in a sinusoidal temporal modulation of the interference intensity. By sampling the interferogram at different time instants, the phase difference between the two beams can be reconstructed. The principle is described as follows.

The electric fields of the signal beam and the reference beam are given by

(4) $$\begin{array}{cc} E_{s}\left(x,y,t\right) & =A_{s}\left(x,y\right)\exp\left\{j\left[2\pi f_{s}t+\phi_{s}\left(x,y\right)\right]\right\},\\ E_{r}\left(x,y,t\right) & =A_{r}\left(x,y\right)\exp\left\{j\left[2\pi f_{r}t+\phi_{r}\left(x,y\right)\right]\right\}, \end{array}$$

where $A_{s}$ and $A_{r}$ denote the field amplitudes of the signal and reference beams, respectively. $f_{s}$ and $f_{r}$ represent their optical frequencies. $\Delta f=f_{s}-f_{r}$ is the heterodyne frequency shift introduced by the AOMs, and $\phi_{s}\left(x,y\right)$ and $\phi_{r}\left(x,y\right)$ are the corresponding wavefront phases.

The detected interferogram intensity is given by the squared magnitude of the superposed fields:

(5) $$I\left(x,y,t\right)=|E_{s}+E_{r}{|}^{2}=A_{s}^{2}+A_{r}^{2}+2A_{s}A_{r}\cos\left[\phi \left(x,y\right)+2\pi \Delta ft\right],$$

which can be rewritten as

(6) $$I\left(x,y,t\right)=I_{0}\left(x,y\right)+I_{m}\left(x,y\right)\cos\left[\phi \left(x,y\right)+2\pi \Delta ft\right],$$

where $I_{0}=A_{s}^{2}+A_{r}^{2}$, $I_{m}=2A_{s}A_{r}$, and $\phi \left(x,y\right)=\phi_{s}\left(x,y\right)-\phi_{r}\left(x,y\right)$.

It can be seen from the above equation that the desired phase information, ϕ(x,y), is contained in the interference signal and undergoes a linear phase shift with time. Therefore, by recording the phase-shifted interferograms at different moments, ϕ(x,y) can be retrieved.

In comparison, mechanical phase shifting changes the phase difference between the reference beam and the measurement beam using a piezoelectric transducer (PZT). However, PZT-based phase shifting usually requires additional compensation and calibration due to mechanical nonlinearities, creep, and hysteresis effects. Heterodyne phase shifting can provide temporally continuous and stable phase-shifted interferograms without any mechanical movement, resulting in a more stable system configuration [22].

To retrieve ϕ(x,y), the interferogram is sampled at discrete time instants $t_{k}$(k=0,1,2,…,K−1), yielding

(7) $$I_{k}\left(x,y\right)=I_{0}\left(x,y\right)+I_{m}\left(x,y\right)\cos\left[\phi \left(x,y\right)+\delta_{k}\right],$$

where $\delta_{k}=2\pi \Delta ft_{k}$ denotes the phase shift at time $t_{k}$. Since the system contains *K* equations with three unknowns, it becomes an overdetermined system when K>3, which can be solved using the least-squares method. To avoid nonlinear optimization, the expression is linearized for parameter estimation using linear least squares.

The linearized form can be written as

(8) $$I_{k}=I_{0}+I_{m}\cos\phi \cos\delta_{k}-I_{m}\sin\phi \sin\delta_{k}=D_{0}+D_{1}\cos\delta_{k}+D_{2}\sin\delta_{k},$$

where $D_{0}=I_{0}$, $D_{1}=I_{m}\cos\phi$, and $D_{2}=I_{m}\sin\phi$.

The parameters $D_{0}$, $D_{1}$, and $D_{2}$ can be obtained as

(9) $${\left[\begin{array}{ccc} D_{0} & D_{1} & D_{2} \end{array}\right]}^{T}={\left(A^{T}A\right)}^{-1}A^{T}I,$$

where

(10) $$A=\left[\begin{array}{ccc} 1 & \cos\delta_{0} & \sin\delta_{0}\\ 1 & \cos\delta_{1} & \sin\delta_{1}\\ \vdots & \vdots & \vdots \\ 1 & \cos\delta_{K-1} & \sin\delta_{K-1} \end{array}\right],\;I=\left[\begin{array}{c} I_{0}\\ I_{1}\\ \vdots \\ I_{K-1} \end{array}\right].$$

When the interferogram is sampled uniformly over one heterodyne period, i.e., $\delta_{k}=2\pi k/K$, Equation (9) can be further simplified as

(11) $${\left[\begin{array}{ccc} D_{0} & D_{1} & D_{2} \end{array}\right]}^{T}={\left[\begin{array}{ccc} \frac{1}{K}\sum_{k=0}^{K-1}I_{k} & \frac{2}{K}\sum_{k=0}^{K-1}I_{k}\cos\delta_{k} & \frac{2}{K}\sum_{k=0}^{K-1}I_{k}\sin\delta_{k} \end{array}\right]}^{T}.$$

Therefore, the phase difference between the signal beam and the reference beam can be expressed as

(12) $$\phi =-\tan^{-1}\left(\frac{D_{2}}{D_{1}}\right)=-\tan^{-1}\left(\frac{\sum_{k=0}^{K-1}I_{k}\sin\delta_{k}}{\sum_{k=0}^{K-1}I_{k}\cos\delta_{k}}\right).$$

Then, the phase can be calculated according to Equation (12), and is computed independently for each pixel. Since the obtained phase distribution lies within [−π,π], phase unwrapping is required. A branch-cut method is adopted in this work, which has a computational complexity of O(N), where *N* is the number of pixels. The algorithm effectively avoids unwrapping errors caused by phase noise by operating in linear time. The procedure consists of the following: (1) detecting residue points using contour integration; (2) constructing branch cuts based on the residues and (3) performing unwrapping via a breadth-first search strategy that avoids crossing branch cuts.

## 3. System Error Simulation and Analysis

The system errors in DOF-HPSPDI mainly originate from two sources. The first arises from the oblique cleaving of the fiber, which introduces wavefront errors in the point diffraction wavefront; this effect is analyzed in detail in Section 3.1. The second is caused by the spatial mismatch between the emission points of the reference beam and the measurement beam, which is discussed in Section 3.2.

### 3.1. Point Diffraction Wavefront Error Induced by Oblique Fiber Cleaving

Oblique cleaving of the fiber causes the emitted field to deviate from an ideal circular Gaussian beam, thereby distorting the phase distribution of the diffracted wavefront in the far field. To quantitatively investigate the influence of the cleaving angle on the point diffraction wavefront, the following computational model is established.

**Figure 5:**
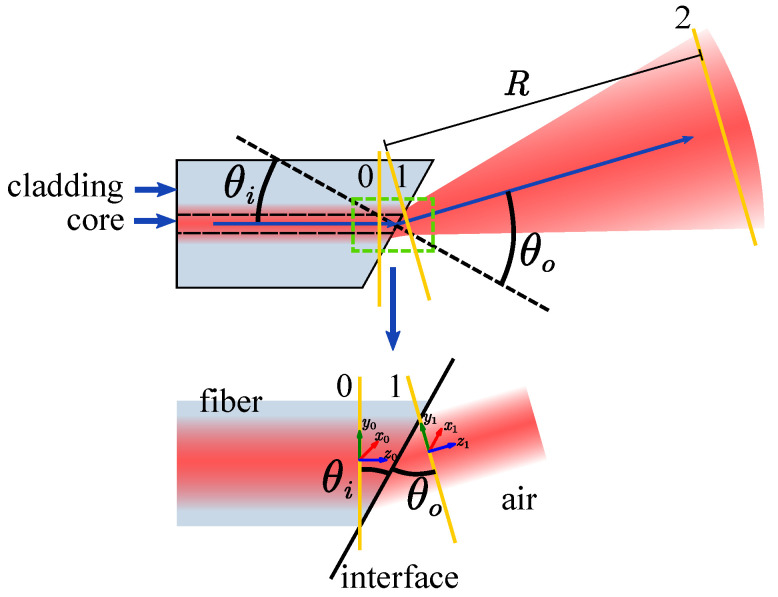
Schematic of the point diffraction wavefront model for an obliquely cleaved fiber.

As shown in Figure 5, the optical field on an arbitrary cross-section (Plane 0) inside the fiber can be approximated by a Gaussian distribution:

(13) $$E_{0}\left(x_{0},y_{0}\right)=A_{0}\exp\left(-\frac{x_{0}^{2}+y_{0}^{2}}{\omega_{0}^{2}}\right).$$

where $\omega_{0}$ is the Gaussian beam waist radius, defined as half of the mode field diameter (MFD) of the fiber, based on the $1/e^{2}$ intensity criterion.

According to Gaussian beam propagation theory, within a small region around the beam waist, the wavefront can be approximated as locally planar. After refraction at the obliquely cleaved fiber end-face, the propagation direction of the beam changes. The mapping between a point on Plane 0 and its corresponding point on the exit surface (Plane 1) can be expressed as follows:

(14) $$x_{1}=x_{0},\;\frac{y_{1}}{\cos\theta_{o}}=\frac{y_{0}}{\cos\theta_{i}}.$$

Therefore, the optical field distribution on the exit surface (Plane 1) becomes the following:

(15) $$E_{1}\left(x_{1},y_{1}\right)=A_{0}\exp\left[-\frac{x_{1}^{2}+{\left(\frac{\cos\theta_{i}}{\cos\theta_{o}}\right)}^{2}y_{1}^{2}}{\omega_{0}^{2}}\right],$$

which can be rewritten as an elliptical Gaussian form:

(16) $$E_{1}\left(x_{1},y_{1}\right)=A_{0}\exp\left(-\frac{x_{1}^{2}}{\omega_{0x}^{2}}-\frac{y_{1}^{2}}{\omega_{0y}^{2}}\right),$$

where

(17) $$\omega_{0x}=\omega_{0},\;\omega_{0y}=\frac{\cos\theta_{o}}{\cos\theta_{i}}\;\omega_{0}.$$

It can be seen that the refraction induces compression of the beam along the *y*-direction, transforming the initially circularly symmetric Gaussian beam into an elliptical Gaussian beam. According to Gaussian beam propagation properties, the direction with a smaller beam waist corresponds to a larger divergence angle.

To calculate the diffracted field on an observation plane (Plane 2) located at a distance *R* from the fiber end-face, the Rayleigh–Sommerfeld diffraction integral of the first kind is employed:

(18) $$E_{2}\left(x_{2},y_{2}\right)=\frac{1}{i\lambda }\;\int \int \;E_{1}\left(x_{1},y_{1}\right)\frac{e^{ikr}}{r}\cos\theta \;dx_{1}dy_{1},$$

where

(19) $$r=\sqrt{{\left(x_{2}-x_{1}\right)}^{2}+{\left(y_{2}-y_{1}\right)}^{2}+R^{2}},$$

represents the distance between a point $\left(x_{1},y_{1}\right)$ on the exit surface and a point $\left(x_{2},y_{2}\right)$ on the observation plane. The angle θ is defined as the angle between the propagation direction and the surface normal, with cosθ=R/r.

It should be noted that no Fresnel or Fraunhofer approximation is introduced in this model, making it applicable to arbitrary propagation distances and angles.

The phase and intensity distributions on Plane 2 are given by the following:

(20) $$\phi_{2}\left(x_{2},y_{2}\right)=\mathrm{Unwrap}\left(\arg\left[E_{2}\left(x_{2},y_{2}\right)\right]\right),$$

(21) $$I_{2}\left(x_{2},y_{2}\right)={\left|E_{2}\left(x_{2},y_{2}\right)\right|}^{2}.$$

where Unwrap(·) denotes the phase unwrapping operation.

Finally, the deviation of the wavefront from an ideal spherical wavefront (in units of wavelength λ) is defined as follows:

(22) $$\Delta W\left(x_{2},y_{2}\right)=\frac{\phi_{2}\left(x_{2},y_{2}\right)}{2\pi }-\frac{\sqrt{x_{2}^{2}+y_{2}^{2}+R^{2}}}{\lambda }.$$

Based on the above model, we simulated the complex amplitude distributions on the fiber exit surface (Plane 1) and on the observation plane at a distance of R=100 mm (Plane 2) within a numerical aperture of NA=0.12, for obliquely cleaved fiber angles of $0^{\circ }$, $29^{\circ }$, and $36^{\circ }$. The corresponding wavefront deviations from an ideal spherical wavefront on Plane 2 were also calculated. The fiber used in the experiments was a PM630-HP fiber, with a mode field diameter (MFD) of 4.5 μm.

**Figure 6:**
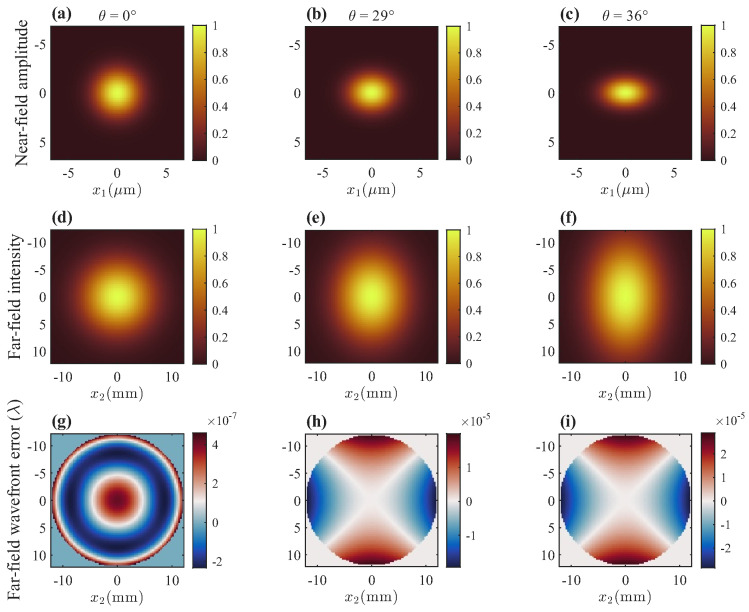
Effects of fiber cleaving angle on the amplitude and phase distributions of the fiber output, for an observation plane at R=100 mm within NA=0.12. Panels (**a**–**c**) show the amplitude distributions on Plane 1 for cleaving angles of $0^{\circ }$, $29^{\circ }$, and $36^{\circ }$, respectively. Panels (**d**–**f**) show the intensity distributions on Plane 2 for the same three cleaving angles. Panels (**g**–**i**) present the wavefront deviations from an ideal spherical wavefront on Plane 2 for the same three cleaving angles.

The results are shown in Figure 6. Panels (a–c), (d–f), and (g–i) correspond to the three cleaving angles, displaying, respectively, the amplitude distributions on Plane 1, the intensity distributions on Plane 2, and the wavefront errors on Plane 2 (in units of wavelength λ).

It can be observed that, as the cleaving angle increases, the beam on Plane 1 is gradually compressed along the *y*-direction, resulting in an increased divergence angle along *y* and a corresponding elongation of the beam along *y* on Plane 2. Meanwhile, the wavefront deviations on Plane 2 exhibit an astigmatic pattern, characteristic of the point diffraction wavefront from an obliquely cleaved fiber.

**Figure 7:**
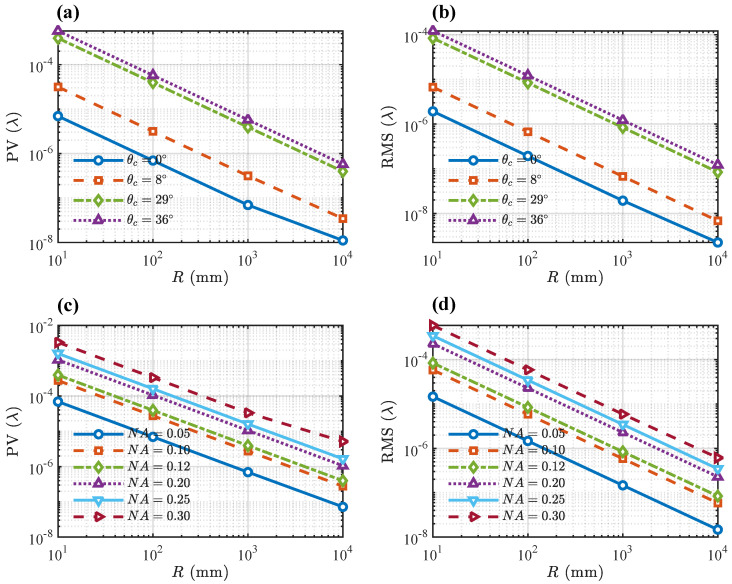
PV and RMS wavefront errors on the far-field observation plane at different distances *R*. (**a**) PV wavefront errors as a function of *R* for fibers with different cleaving angles within NA=0.12. (**b**) RMS wavefront errors as a function of *R* for fibers with different cleaving angles within NA=0.12. (**c**) PV wavefront errors as a function of *R* for a cleaving angle of $29^{\circ }$ under different NAs. (**d**) RMS wavefront errors as a function of *R* for a cleaving angle of $29^{\circ }$ under different NAs.

The wavefront error is influenced by the fiber cleaving angle $\theta_{c}$, the distance *R* to the observation plane, and the observation numerical aperture (NA). Figure 7a,b show the peak-to-valley (PV) and root-mean-square (RMS) wavefront errors as functions of *R* for different cleaving angles, within an observation NA of 0.12. Figure 7c,d present the PV and RMS wavefront errors versus *R* for a cleaving angle of $29^{\circ }$ under different NAs.

It can be concluded that the wavefront error decreases with increasing *R*, and increases with increasing cleaving angle $\theta_{c}$ and NA.

For the fiber used in the experiments (PM630-HP) with a cleaving angle of $29^{\circ }$ and NA = 0.12, the wavefront PV and RMS errors are better than $3.9\times 10^{-4}\;\lambda$ and $8.4\times 10^{-5}\;lambda$, respectively, for R>10 mm. At R>100 mm, the PV and RMS errors improve to better than $3.9\times 10^{-5}\;\lambda$ and $8.4\times 10^{-6}\;\lambda$, respectively.

**Figure 8:**
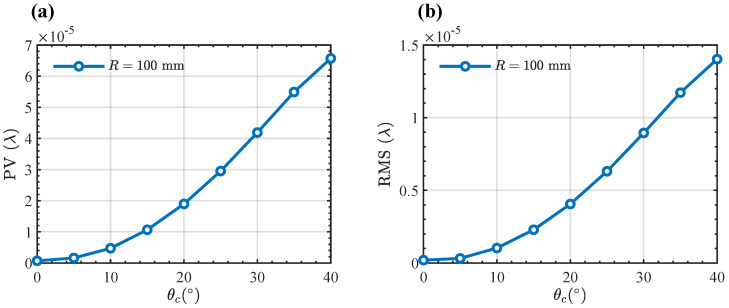
PV and RMS wavefront errors on the far-field observation plane at different cleaving angles $\theta_{c}$ within NA=0.12 and R=100 mm. (**a**) PV wavefront errors as a function of $\theta_{c}$ within NA=0.12 and R=100 mm. (**b**) RMS wavefront errors as a function of $\theta_{c}$ within NA=0.12 and R=100 mm.

Figure 8 illustrates the relationship between the cleaving angle and the quality of the point-diffracted wavefront. It can be observed that, even at relatively large cleaving angles, although the wavefront quality slightly degrades, the magnitude of the degradation remains very small and is still sufficient for high-precision wavefront measurements.

### 3.2. System Error Induced by Non-Coincident Diffraction Points of the Reference and Measurement Beams

**Figure 9:**
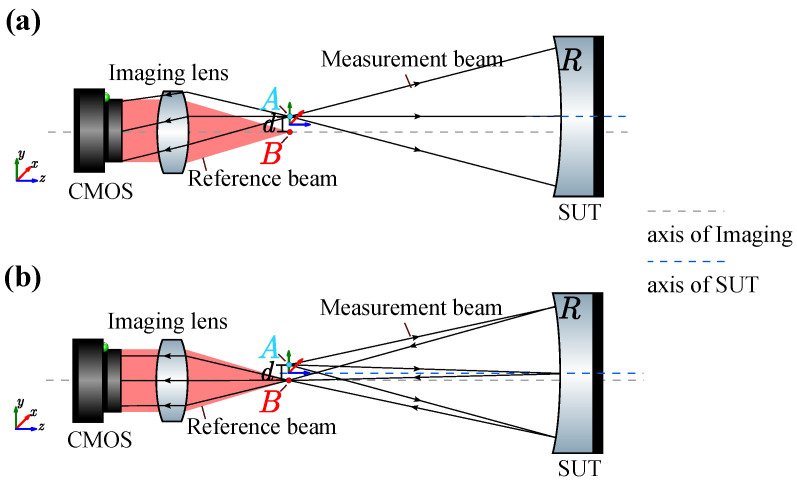
Schematic illustration of errors caused by non-coincident diffraction points of the measurement and reference beams. (**a**) The measurement beam retraces its original path. (**b**) The measurement beam returns to the reference beam position.

As shown in Figure 9, the error induced by the non-coincident diffraction points of the measurement and reference beams is illustrated, where the folded optical path is unfolded for clarity. As shown in Figure 9a, when the diffraction point A of the reference beam and the diffraction point B of the measurement beam do not coincide, if the measurement beam retraces its original path, the two beams propagate along different paths within the imaging system, resulting in unknown return-path errors introduced by the system.

To overcome this issue, the configuration shown in Figure 9b is adopted. In this case, the measurement beam emitted from diffraction point A is reflected by the SUT and converges onto the reference diffraction point B. After that, it shares the same optical path as the reference beam in the subsequent propagation.

This strategy eliminates the unknown return-path errors introduced by the imaging system. The measurement error introduced in this process is purely geometric and can be analytically calculated. Therefore, the system error can be directly compensated without additional calibration. In the following, the magnitude of this system error is derived.

For an arbitrary point P(x,y,z) on the surface under test (SUT) with a radius of curvature *R*, the following relation holds:

(23) $$\sqrt{x^{2}+y^{2}+z^{2}}=R.$$

The optical path length from the reference diffraction point to the measurement diffraction point via point *P* is given by the following:

(24) $$S\left(x,y\right)=\sqrt{x^{2}+{\left(y-d/2\right)}^{2}+z^{2}}+\sqrt{x^{2}+{\left(y+d/2\right)}^{2}+z^{2}},$$

where *d* is the separation between the two fiber emission points.

By substituting $x^{2}+y^{2}+z^{2}=R^{2}$, the expression can be rewritten as follows:

(25) $$S\left(x,y\right)=\sqrt{R^{2}-2yd+\frac{d^{2}}{4}}+\sqrt{R^{2}+2yd+\frac{d^{2}}{4}}.$$

Using the second-order Taylor expansion:

(26) $$\sqrt{R^{2}+\varepsilon }=R+\frac{\varepsilon }{2R}-\frac{\varepsilon^{2}}{8R^{3}}+O\left(\varepsilon^{3}\right),$$

we obtain the following:

(27) $$S\left(x,y\right)=2R+\frac{d^{2}}{4R}-\frac{4y^{2}d^{2}+d^{4}}{16R^{3}}.$$

Neglecting higher-order terms beyond $d^{2}$, this simplifies to the following:

(28) $$S\left(x,y\right)=2R+\frac{d^{2}}{4R}-\frac{y^{2}d^{2}}{4R^{3}}.$$

This expression can be decomposed into lower-order aberration terms:

(29) $$S\left(x,y\right)=2R+\frac{d^{2}}{4R}-\frac{\left(x^{2}+y^{2}\right)d^{2}}{8R^{3}}+\frac{\left(x^{2}-y^{2}\right)d^{2}}{8R^{3}}.$$

Since piston and defocus terms do not affect the measurement, the resulting system error corresponds to astigmatism:

(30) $$\Delta S\left(x,y\right)=\frac{d^{2}}{8R^{3}}\left(x^{2}-y^{2}\right).$$

Let the aperture radius be *a*, and define normalized coordinates:

(31) $$x_{0}=\frac{x}{a}=\rho \cos\theta ,\;y_{0}=\frac{y}{a}=\rho \sin\theta ,$$

where (ρ,θ) are normalized polar coordinates with 0≤ρ≤1.

Then, the optical path difference becomes the following:

(32) $$\Delta S\left(x_{0},y_{0}\right)=\frac{d^{2}a^{2}}{8R^{3}}\left(x_{0}^{2}-y_{0}^{2}\right).$$

Since the interferometer is a double-pass system, the actual measurement error should be divided by 2. Normalizing by the wavelength λ, the wavefront error is as follows:

(33) $$\Delta W_{d}\left(x_{0},y_{0}\right)=\frac{d^{2}a^{2}}{16R^{3}\lambda }\left(x_{0}^{2}-y_{0}^{2}\right).$$

By defining the numerical aperture as NA=a/R, the above expression can be rewritten as follows:

(34) $$\Delta W_{d}\left(x_{0},y_{0}\right)=\frac{d^{2}{\mathrm{NA}}^{2}}{16R\lambda }\left(x_{0}^{2}-y_{0}^{2}\right).$$

It can be seen that the measurement error induced by the non-coincident diffraction points manifests as astigmatism. It is proportional to the square of the separation *d*, inversely proportional to the radius of curvature *R*, and proportional to the square of the numerical aperture. By subtracting this error term, the systematic error can be corrected to a certain extent.

Furthermore, this system error is insensitive to the position of the measurement region on the detector, since

(35) $${\left(x+\Delta x\right)}^{2}-{\left(y+\Delta y\right)}^{2}=\left(x^{2}-y^{2}\right)+2x\Delta x-2y\Delta y+\Delta x^{2}-\Delta y^{2}.$$

**Figure 10:**
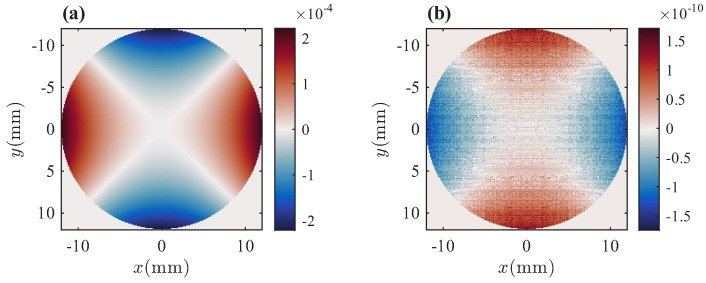
Distribution of the system error $\Delta W_{d}$ (in units of wavelength λ) for an SUT with R=100 mm, NA=0.12, and fiber separation d=125 μm. (**a**) System error without correction. (**b**) Residual error after subtracting the derived analytical result.

Figure 10 illustrates the system error $\Delta W_{d}$ for an SUT with a radius of curvature R=100 mm, a numerical aperture of NA=0.12, and a fiber separation of d=125 μm. Figure 10a shows the distribution of the system error without approximation, yielding a peak-to-valley (PV) value of $4.4\times 10^{-4}\;\lambda$ and a root-mean-square (RMS) value of $8.0\times 10^{-5}\;\lambda$. Figure 10b shows the residual error after subtracting the analytical approximation given by Equation (34), where the PV and RMS values are reduced to $3.5\times 10^{-10}\;\lambda$ and $4.8\times 10^{-11}\;\lambda$, respectively.

These results demonstrate the validity and high accuracy of the derived analytical expression in Equation (34).

**Figure 11:**
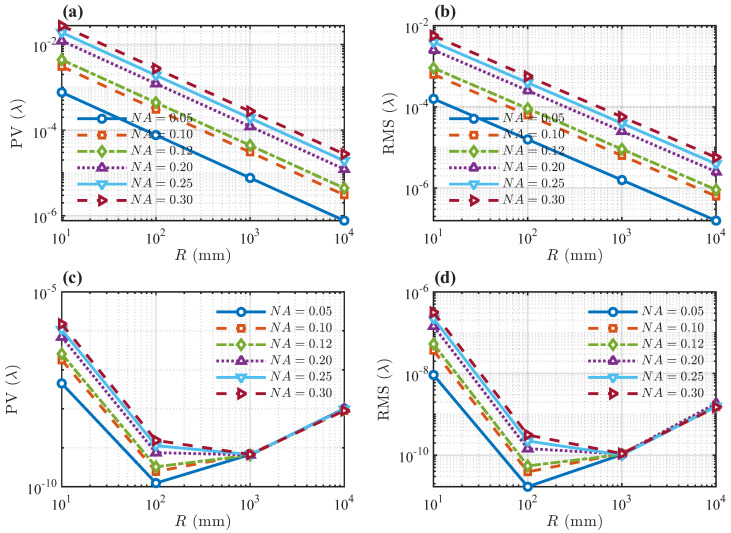
PV and RMS of the system error $\Delta W_{d}$ as functions of the SUT radius of curvature *R* under different numerical apertures (NA), with a fiber separation of d=125 μm. (**a**) PV of the uncorrected system error versus *R* under different NAs. (**b**) RMS of the uncorrected system error versus *R* under different NAs. (**c**) PV of the corrected system error versus *R* under different NAs. (**d**) RMS of the corrected system error versus *R* under different NAs.

Figure 11 shows the dependence of the system error induced by the non-coincident diffraction points on the radius of curvature *R* of the SUT and the numerical aperture (NA), with a fiber separation of d=125 μm.

As shown in Figure 11a,b, the magnitude of the system error is inversely proportional to *R* and increases with increasing NA. Figure 11c,d present the residual error after correction using the derived analytical approximation. It can be seen that, within the measurement range of R=10–10,000 mm and NA<0.3, the peak-to-valley (PV) error is less than $1.5\times 10^{-6}\;lambda$ and the root-mean-square (RMS) error is less than $3.1\times 10^{-7}\;\lambda$. For NA<0.12, the PV and RMS errors are further reduced to below $2.5\times 10^{-7}\;\lambda$ and $5.2\times 10^{-8}\;\lambda$, respectively.

**Figure 12:**
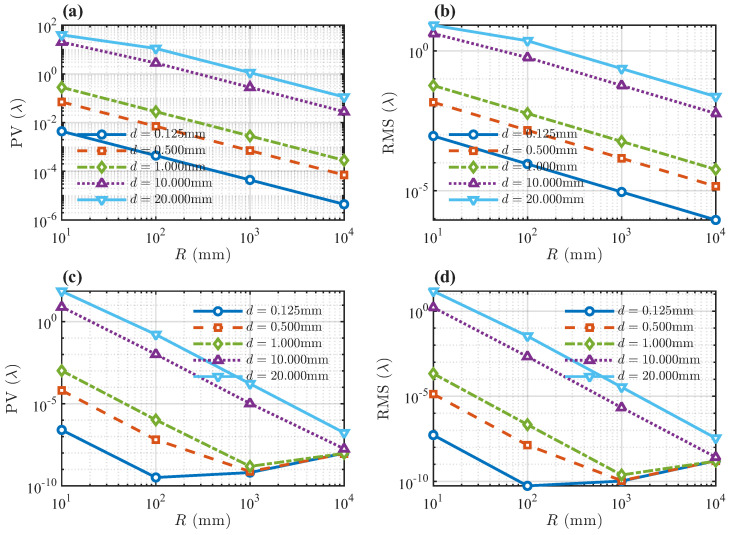
PV and RMS of the system error $\Delta W_{d}$ as functions of the SUT radius of curvature *R* for different fiber separations *d*, with NA=0.12. (**a**) PV of the uncorrected system error versus *R* for different fiber separations. (**b**) RMS of the uncorrected system error versus *R* for different fiber separations. (**c**) PV of the corrected system error versus *R* for different fiber separations. (**d**) RMS of the corrected system error versus *R* for different fiber separations.

Figure 12 illustrates the dependence of the system error caused by non-coincident diffraction points on the SUT radius of curvature *R* and the fiber separation *d*, with the SUT numerical aperture fixed at NA=0.12.

As shown in Figure 12a,b, the system error increases with increasing fiber separation *d*. Figure 12c,d show that for large fiber separations, the system error cannot be effectively reduced to a low level using the derived analytical correction formula.

It can be calculated that, under the condition of NA = 0.12, R = 100 mm, in order to achieve a wavefront PV error smaller than 1/1000 λ, the fiber separation must satisfy d<0.19 mm without correction, while with correction, the allowable separation can be relaxed to d<5.6 mm.

When the fiber separation *d* becomes relatively large, compensation of higher-order terms is indeed necessary. Therefore, in our design, we aim to minimize the fiber separation *d* as much as possible (d = 125 μm), so that the systematic error remains sufficiently small even without correction.

When implementing the error correction, since the system parameters *R*, *d*, and NA may contain uncertainties, errors may also be introduced during the correction process. Let

(36) $$C=\frac{d^{2}{\mathrm{NA}}^{2}}{16R\lambda }$$

Then, the relationship between the uncertainty of *C* and the uncertainties of the corresponding parameters can be obtained as

(37) $$\begin{array}{cc} \frac{u^{d}\left(C\right)}{C} & =2\frac{u(d)}{d},\\ \frac{u^{NA}\left(C\right)}{C} & =2\frac{u(NA)}{NA},\\ \frac{u^{R}\left(C\right)}{C} & =\frac{u(R)}{R}, \end{array}$$

and

(38) $$\begin{array}{cc} \frac{u(C)}{C} & =\sqrt{{\left(\frac{u^{d}\left(C\right)}{u(C)}\right)}^{2}+{\left(\frac{u^{NA}\left(C\right)}{u(C)}\right)}^{2}+{\left(\frac{u^{R}\left(C\right)}{u(C)}\right)}^{2}}\\ \; & =\sqrt{4{\left(\frac{u(d)}{d}\right)}^{2}+4{\left(\frac{u(NA)}{NA}\right)}^{2}+{\left(\frac{u(R)}{R}\right)}^{2}} \end{array}$$

If the relative uncertainties of these parameters are assumed to be uniformly distributed, the following relationship can be obtained:

(39) $$\frac{u(C)}{C}=3\frac{u(p)}{p}$$

where *p* represents any one of these parameters. For the parameters d=125 μm, R=100mm, and NA=0.12, the corresponding systematic error is $\mathrm{PV}=4.4\times 10^{-4}\;\lambda$. If the relative uncertainty satisfies $\frac{u(p)}{p}=0.01$, the residual correction error is $\mathrm{PV}=1.3\times 10^{-5}\;\lambda$.

The residual correction error is related not only to the uncertainties of the parameters but also to the magnitude of the systematic error itself. Therefore, by minimizing fiber spacing *d*, the residual correction error can also be effectively reduced.

## 4. Experimental Setup and Results

**Figure 13:**
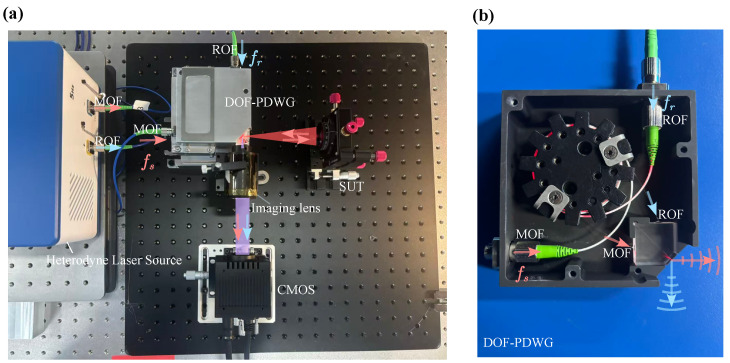
Experimental setup. (**a**) Experimental setup of the DOF-HPSPDI system. (**b**) Detailed structure of the DOF-PDWG.

Figure 13 shows the experimental setup of the DOF-HPSPDI system. A self-developed heterodyne light source with a wavelength of λ=632.8 nm is employed. The heterodyne frequency difference between the two output beams is Δf=5 Hz. These two beams are coupled into the reference optical fiber (ROF) and measurement optical fiber (MOF) of the DOF-PDWG to generate the reference wave and measurement wave, respectively.

The SUT used in the experiment is a concave spherical surface with a radius of curvature of 100 mm and a nominal surface quality of PV 1/10 λ. It was mounted on an adjustment stage to align its position with respect to the system so as to satisfy the condition for null testing.

The CMOS camera operates at a sampling frequency of $f_{s}=100$ Hz, corresponding to 20 interferograms acquired within one heterodyne period. Choosing a higher heterodyne frequency can enhance the suppression of vibration-induced disturbances, while a larger number of phase-shifting steps can improve the suppression of noise [24].

The detailed structure and packaging of the DOF-PDWG are shown in Figure 13b. Both the ROF and MOF are fabricated using PM630HP fibers, with a core diameter of 2a=3.5 μm, cladding diameter of 2b=125 μm, and mode field diameter (MFD) of 4.5 μm. The fiber cleaving angle is $\theta_{c}=29^{\circ }$, and the separation between the two fibers is d=125 μm. The included angle between the reference and measurement beams is $2\theta_{o}=90^{\circ }$.

The main system parameters are summarized in Table 1.

**Figure 14:**
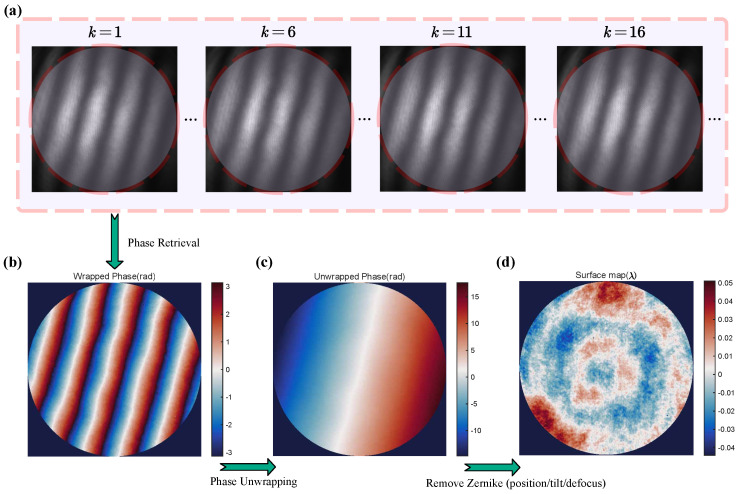
Phase retrieval in heterodyne phase-shifting interferometry. (**a**) Interferograms. (**b**) Wrapped phase. (**c**) Unwrapped phase. (**d**) Reconstructed surface after removing piston, tilt, and defocus.

As shown in Figure 14, the phase retrieval process of heterodyne phase-shifting interferometry is illustrated. Figure 14a presents four representative interferograms selected from the 20-step phase-shifting sequence, corresponding to the 1st, 6th, 11th, and 15th frames. Figure 14b shows the retrieved wrapped phase, which is confined within the range of [−π,π]. Figure 14c displays the unwrapped continuous phase distribution. Figure 14d shows the reconstructed surface after removing piston, tilt, and defocus terms via Zernike polynomial fitting.

**Figure 15:**
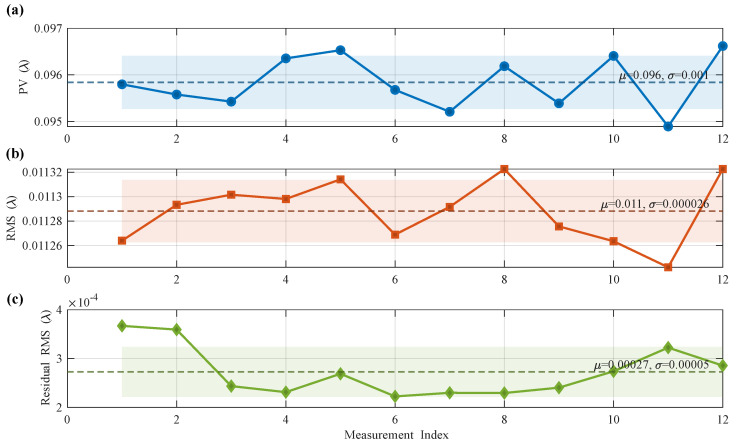
Repeatability results. (**a**) Peak-to-valley (PV) values of the measured surfaces. (**b**) Root-mean-square (RMS) values of the measured surfaces. (**c**) RMS of residuals with respect to the mean surface.

Through repeated measurements, a series of surface maps $W_{i}$ (i=1,2,…,N) are obtained. The repeatability results are shown in Figure 15.

Figure 15a,b show the PV and RMS values obtained from multiple measurements, respectively. The corresponding standard deviations are calculated as

(40) $$\sigma \left(PV\right)=\sqrt{\frac{\sum_{i=1}^{N}{\left(PV_{i}-\bar{PV}\right)}^{2}}{N-1}},\;\sigma \left(RMS\right)=\sqrt{\frac{\sum_{i=1}^{N}{\left(RMS_{i}-\bar{RMS}\right)}^{2}}{N-1}}.$$

The calculated results are σ(PV)=0.001λ and $\sigma \left(RMS\right)=2.6\times 10^{-5}\;\lambda$.

The mean surface is obtained by averaging all measurements:

(41) $$\bar{W}=\frac{1}{N}\sum_{i=1}^{N}W_{i}.$$

The residual between each measurement and the mean surface is defined as

(42) $$W_{i}^{\mathrm{res}}=W_{i}-\bar{W}.$$

The root-mean-square (RMS) of each residual map, denoted as $RMS(W_{i}^{\mathrm{res}})$, is calculated, and the statistical results are shown in Figure 15c. The mean and standard deviation are given by

(43) $$\bar{RMS(W^{\mathrm{res}})}=\frac{1}{N}\sum_{i=1}^{N}RMS\left(W_{i}^{\mathrm{res}}\right),$$

(44) $$\sigma \left(RMS\left(W^{\mathrm{res}}\right)\right)=\sqrt{\frac{\sum_{i=1}^{N}{\left(RMS\left(W_{i}^{\mathrm{res}}\right)-\bar{RMS(W^{\mathrm{res}})}\right)}^{2}}{N-1}}.$$

The calculated results are $\bar{RMS(W^{\mathrm{res}})}=0.00027\;\lambda$ and $\sigma \left(RMS\left(W^{\mathrm{res}}\right)\right)=5\times 10^{-5}\;\lambda$.

## 5. Discussion

### 5.1. System Performance

According to the analysis in Section 3.1, for the system described in Section 4, the peak-to-valley (PV) value of the wavefront generated by the oblique fiber point diffraction is better than $3.9\times 10^{-5}\;\lambda$, and the root-mean-square (RMS) value is better than $8.4\times 10^{-6}\;\lambda$.

Based on the analysis in Section 3.2, without applying system error correction, the measurement error caused by the mismatch between the two diffraction points is less than $4.4\times 10^{-4}\;\lambda$ in PV and $8\times 10^{-5}\;\lambda$ in RMS. After compensating for the system error, and considering a 1% uncertainty in the correction parameters, the residual systematic error can be corrected to the level of $\mathrm{PV}=1.3\times 10^{-5}\;\lambda$ and $\mathrm{RMS}=2.4\times 10^{-6}\;\lambda$.

The feasibility and stability of the proposed system are experimentally verified. The measurement stability is quantitatively evaluated using the RMS and the residual RMS, denoted as $RMS(W^{\mathrm{res}})$. The measurement uncertainty of the RMS, expressed as the 2σ value (corresponding to approximately a 95% confidence interval), is given by

(45) $$2\sigma \left(RMS\right)=5.2\times 10^{-5}\;\lambda ,$$

which corresponds to 0.0329nm, also referred to as RMS simple repeatability.

Furthermore, by analyzing the mean and standard deviation of $RMS(W^{\mathrm{res}})$, the deviation of a single measurement from the mean surface can be evaluated. The statistical upper bound (at the 2σ level) can be expressed as

(46) $$\bar{RMS(W^{\mathrm{res}})}+2\sigma \left(RMS\left(W^{\mathrm{res}}\right)\right)=3.7\times 10^{-4}\;\lambda ,$$

which corresponds to 0.2341nm, also referred to as RMS wavefront repeatability.

These results demonstrate that the proposed system achieves high repeatability and stability, enabling sub-nanometer-level surface measurement accuracy.

### 5.2. Influence of Oblique-Incidence Measurement

**Figure 16:**
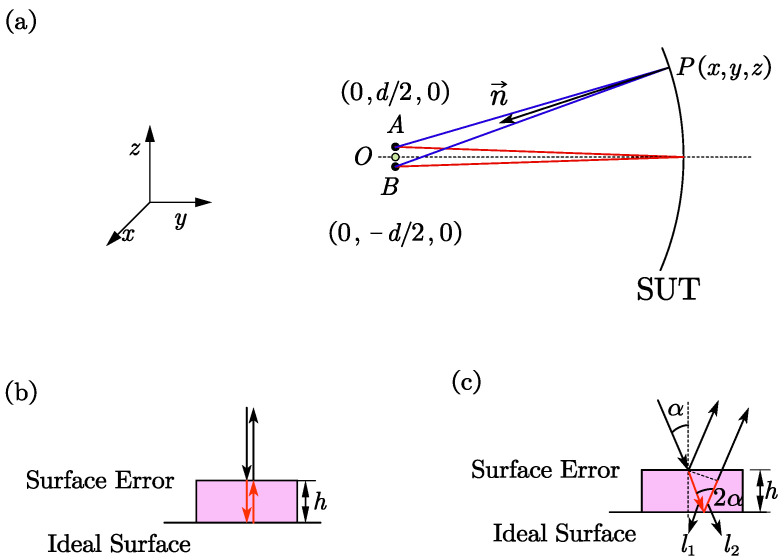
Schematic diagram of the influence of oblique-incidence measurement. (**a**) The configuration of the proposed system. (**b**) Incident along the surface normal. (**c**) Incident at a certain angle relative to the surface normal.

As stated in Section 3.2, in our configuration, the light is incident obliquely on the surface under test, i.e., it deviates slightly from normal incidence, as shown in the Figure 16a.

For an arbitrary point P(x,y,z) on the surface under test, the angle α between the incident beam and the surface normal can be expressed as

(47) $$\cos\alpha =\frac{\vec{OP}\cdot \vec{AP}}{|\vec{OP}\left|\;\right|\vec{AP}|}$$

where

(48) $$\vec{OP}=\left(x,y,z\right)$$

(49) $$\vec{AP}=\left(x,y-\frac{d}{2},z\right)$$

When the measurement beam is incident along the surface normal, the optical path difference is 2h, as shown in Figure 16a.When the measurement beam forms an angle α with respect to the surface normal of the surface under test, as shown in Figure 16b, the optical path difference becomes

(50) $$L=l_{1}+l_{2}$$

where

(51) $$l_{1}=\frac{h}{\cos\alpha }$$

(52) $$l_{2}=l_{1}\cos2\alpha$$

Therefore,

(53) $$\begin{array}{cc} L & =l_{1}+l_{2}\\ \; & =l_{1}\left(1+\cos2\alpha \right)\\ \; & =2l_{1}\cos^{2}\alpha \\ \; & =2h\cos\alpha \end{array}$$

Therefore, the measurement sensitivity is related to cosα. A qualitative analysis of the relationship between cosα and 1 can be expressed as

(54) $$1-\cos\alpha =2\sin^{2}\left(\frac{\alpha }{2}\right)\approx \frac{\alpha^{2}}{2}$$

Therefore, when α is very small, the deviation of cosα from 1 is a second-order small quantity and can generally be neglected. In the following, the magnitude of α in the proposed system is further evaluated.

**Figure 17:**
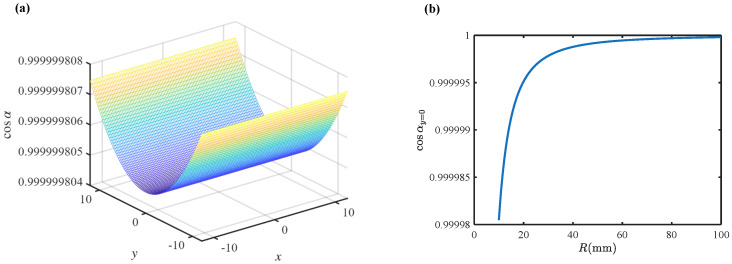
Influence of oblique-incidence measurement (**a**) cosα at different positions on the surface under test for R=100 mm. (**b**) Relationship between $\cos\alpha_{\left(y=0\right)}$ and the radius of curvature *R*.

For NA=0.12, R=100mm, and a fiber spacing of d=125 μm, the calculated values of cosα at different positions on the surface under test are shown in the Figure 17a.

It can be observed that cosα is approximately equal to 1 over the entire surface and increases with increasing *y*. In other words, the maximum deviation from 1 occurs at y=0. Therefore, we further plotted the variation of $\cos\alpha_{\left(y=0\right)}$ as a function of the radius of curvature *R* as shown in the Figure 17b.

Based on the above analysis, it can be concluded that, because the spacing between the two fibers is sufficiently small, the oblique incidence angle is also sufficiently small. Consequently, the sensitivity nonuniformity caused by oblique incidence can be neglected.

### 5.3. Effect of Heterodyne Frequency Drift

**Figure 18:**
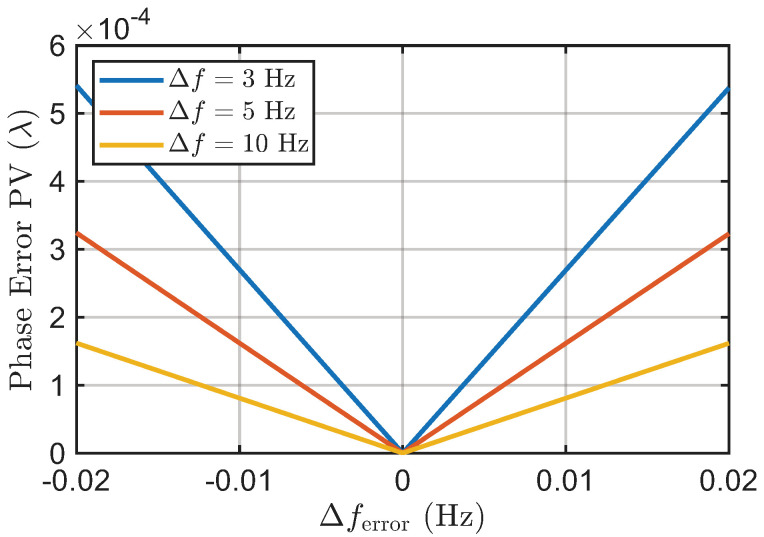
Influence of Heterodyne Frequency Error on Phase Retrieval Accuracy.

A slight drift in the heterodyne frequency, Δf, may affect the accuracy of phase retrieval. We analyzed the influence of heterodyne frequency errors on phase retrieval accuracy under different heterodyne frequencies, as shown in the Figure 18.

### 5.4. Other Influencing Factors

For the proposed DOF-HPSPDI, this work specifically analyzes two major error sources associated with the DOF-PDWG: the wavefront error introduced by the oblique-cleaved fiber point diffraction wavefront and the error caused by the non-coincidence of the two diffraction points.

In addition, as a precision measurement instrument, the system is also subject to many other sources of error, which may affect both the measurement repeatability and the absolute measurement accuracy.

Regarding repeatability, factors such as the stability of the laser power and wavelength, the stability of the heterodyne frequency, and detector noise can all influence the repeatability of the system. Furthermore, environmental disturbances, including atmospheric turbulence and temperature fluctuations, may also degrade the measurement repeatability. Therefore, environmental control is essential for high-precision measurements.

We summarize the estimated contributions of various factors affecting measurement repeatability in Table 2.

What’s more, including fiber cleaving quality, adhesive stress, thermal drift, and alignment sensitivity may indeed affect the absolute measurement accuracy. Among these factors, we believe that fiber cleaving quality and adhesive stress are likely to have the greatest influence, since they may directly affect the quality of the point-diffracted wavefront.

The validation of the absolute measurement accuracy of the system is of great importance [28,29,30,31]. In future work, we plan to adopt the rotation–translation method, which separates the surface figure error of the test optic from the system error through multiple rotations and translations of the specimen. In this way, the system error can be calibrated and the absolute accuracy of the proposed method can be further verified.

Therefore, our future work will focus on the evaluation and optimization of the fabrication quality of the point diffraction generator. By experimentally characterizing both the accuracy of the generated point-diffracted wavefront and the absolute measurement accuracy of the system, we aim to further optimize the fabrication process of the point diffraction generator.

### 5.5. Discussion on Application Scope

The proposed point diffraction interferometer is not only applicable to concave spherical surface testing, but can also be used for transmitted wavefront measurements of optical systems. For high-NA concave surfaces and optical systems, the proposed configuration can be combined with subaperture stitching techniques to extend the measurable numerical aperture range while maintaining high measurement accuracy. For convex surfaces and flat surfaces, the diverging point diffraction reference wave needs to be converted into a converging or collimated wavefront, resulting in a non-common-path interferometric configuration. In such cases, if absolute measurements are required, the systematic errors of the interferometer should be calibrated in advance.

## 6. Conclusions

This paper proposes a dual-oblique-fiber heterodyne phase-shifting point diffraction interferometer (DOF-HPSPDI) and designs a compact dual-oblique-fiber point diffraction wavefront generator (DOF-PDWG). The proposed configuration achieves effective separation of the measurement and reference beams through oblique fibers, while the reflective structure at the fiber end faces enables common-path propagation of the two beams in the imaging system, thereby significantly reducing non-common-path errors. Furthermore, by minimizing the spacing between the two fibers, system errors induced by source mismatch are effectively suppressed. In addition, heterodyne phase-shifting interferometry is employed to achieve stable phase modulation and high-precision wavefront phase reconstruction.

The deviation of the point diffraction wavefront generated by the oblique fiber and the system errors caused by incomplete overlap of the two fibers are theoretically analyzed and numerically simulated, and corresponding error correction methods are proposed. Experimental results verify the feasibility and stability of the proposed system, demonstrating its strong potential for high-precision optical metrology.

This work provides a low-cost, compact, and highly stable point diffraction interferometer, offering a promising solution for future applications in high-precision optical testing and sub-aperture stitching of large-aperture optical components.

In addition, the compact coherent architecture of the proposed system may also provide potential compatibility with future integrated photonic implementations [32,33,34,35].

## Figures and Tables

**Table 1:** System parameters.

Component	Parameter	Value
Heterodyne source	Wavelength λ	632.8 nm
	Frequency difference Δf	5 Hz
DOF-PDWG	Fiber core diameter 2a	3.5 μm
	Fiber cladding diameter 2b	125 μm
	Fiber mode field diameter (MFD)	4.5 μm
	Fiber spacing *d*	125 μm
	Fiber cleaving angle $\theta_{c}$	$29^{\circ }$
	Angular separation between two beams $2\theta_{o}$	$90^{\circ }$
CMOS	Resolution	2048×2048
	Sampling frequency $f_{s}$	100 Hz

**Table 2:** Evaluation of error sources and their contributions to measurement repeatability.

Error Source	Setting Value	RMS Simple Repeatability (λ)	RMS Wavefront Repeatability (λ)
Laser power fluctuation	±0.1%	$0.8\times 10^{-5}$	$2.75\times 10^{-5}$
Laser wavelength error	±0.1 nm	$0.011\times 10^{-5}$	$0.033\times 10^{-5}$
Detector noise	±3 DN @ 8-bit	$0.70\times 10^{-5}$	$1.00\times 10^{-5}$
Heterodyne frequency error	±0.02 Hz	$0.90\times 10^{-5}$	$2.89\times 10^{-5}$
Environmental fluctuations	Temperature: ±0.005 °C; Pressure: ±0.1 Pa; Water vapor pressure: ±5 Pa	$0.8\times 10^{-5}$	$2.58\times 10^{-5}$

## Data Availability

The data related to this study can be requested from the corresponding authors.

## Abbreviations

The following abbreviations are used in this manuscript:

AOM	Acousto-optic modulator
DOF-HPSPDI	Dual-oblique-fiber heterodyne phase-shifting point diffraction interferometer
DOF-PDWG	Dual-oblique-fiber point diffraction wavefront generator
HPSI	Heterodyne phase-shifting interferometry
MOF	Measurement oblique fiber
PDI	Point diffraction interferometry
PSI	Phase-shifting interferometry
ROF	Reference oblique fiber
SMF	Single-mode fiber
SUT	Surface under test

## References

[1] Shan S., Zhao F., Li Z., Luo L., Li X. (2025). A Comprehensive Review of Optical Metrology and Perception Technologies. Sensors.

[2] Wang S., Zheng Z., Zhu W., Duan B., Ju Z.Z., Ju B. (2025). Development of the Stitching—Oblique Incidence Interferometry Measurement Method for the Surface Flatness of Large-Scale and Elongated Ceramic Parts. Sensors.

[3] Ye R., Zeng J., Zhang H., Su Y., Li H. (2025). Rapid Full-Field Surface Topography Measurement of Large-Scale Wafers Using Interferometric Imaging. Photonics.

[4] Zhao F., Tang H., Zou X., Li X. (2025). A Review of Optical Metrology Techniques for Advanced Manufacturing Applications. Micromachines.

[5] Malacara D. (2007). Optical Shop Testing.

[6] Wang D., Xu Y., Liang R., Kong M., Zhao J., Zhang B., Li W. (2016). High-Precision Method for Submicron-Aperture Fiber Point-Diffraction Wavefront Measurement. Opt. Express.

[7] Hibino K., Hanayama R., Kim Y. (2016). Absolute Interferometric Test for High Numerical-Aperture Spherical Concave Surfaces: Gravitational Effect. Measurement.

[8] Xue S., Mao Y., Liu Y., Hu J. (2025). Absolute Test for Flat Surfaces by Shifted and Rotated Maps with Yaw Angle Monitored. Opt. Lett..

[9] Liu R., Ji B., Deng W., Xu Q., Zhang L. (2025). From Theory to Application: Fast, Universal, and Cost-Effective Pixel-Level Surface Reconstruction in Shift-Rotation Absolute Testing. Opt. Express.

[10] Zhai D., Chen S., Peng X., Tie G. (2019). Absolute Flat Test Using Rotated and Multi-Shifted Maps with Relative Tilt Measurement. Opt. Lasers Eng..

[11] Kawashima N., Kondo Y., Hirai A., Bitou Y. (2025). Novel Analysis of Alignment Error on Spherical Fizeau Interferometer and Uncertainty Evaluation of Sphericity Calibration System Based on Random Ball Test. Opt. Lasers Eng..

[12] Smartt R.N., Steel W.H. (1975). Theory and Application of Point-Diffraction Interferometers. Jpn. J. Appl. Phys..

[13] Sommargren G.E., Phillion D.W., Johnson M.A., Nguyen N.Q., Barty A., Snell F.J., Dillon D.R., Bradsher L.S., Engelstad R.L. (2002). 100-Picometer Interferometry for EUVL. Emerging Lithographic Technologies VI.

[14] Sommargren G.E. (1996). Phase Shifting Diffraction Interferometry for Measuring Extreme Ultraviolet Optics. Extreme Ultraviolet Lithography.

[15] Kihm H., Kim S.W., Osten W., Kujawinska M., Creath K. (2003). Oblique Point-Diffraction Source for Interferometer Design. Optical Measurement Systems for Industrial Inspection III.

[16] Kihm H. (2005). Oblique Fiber Optic Diffraction Interferometer for Testing Spherical Mirrors. Opt. Eng..

[17] Matsuura T., Okagaki S., Nakamura T., Oshikane Y., Inoue H., Nakano M., Kataoka T. (2007). Measurement Accuracy in Phase-Shifting Point Diffraction Interferometer with Two Optical Fibers. Opt. Rev..

[18] Matsuura T., Okagaki S., Oshikane Y., Inoue H., Nakano M., Kataoka T. (2008). Numerical Reconstruction of Wavefront in Phase-shifting Point Diffraction Interferometer by Digital Holography. Surf. Interface Anal..

[19] Matsuura T., Udaka K., Oshikane Y., Inoue H., Nakano M., Yamauchi K., Kataoka T. (2010). Spherical Concave Mirror Measurement by Phase-Shifting Point Diffraction Interferometer with Two Optical Fibers. Nucl. Instrum. Methods Phys. Res. Sect. Accel. Spectrometers Detect. Assoc. Equip..

[20] Fen G., Zhao S., He Y., Shi P., Wang T. (2026). Point Diffraction Interferometry System Based on Pinhole-Plate Phase-Shifting. Opt. Laser Technol..

[21] Le Clerc F., Collot L., Gross M. (2000). Numerical Heterodyne Holography with Two-Dimensional Photodetector Arrays. Opt. Lett..

[22] Atlan M., Gross M., Absil E. (2007). Accurate Phase-Shifting Digital Interferometry. Opt. Lett..

[23] Wu Z., Zhang W., Bin X., Kong X., Lehmann P., Osten W., Albertazzi Gonçalves A. (2017). Full-Field Heterodyne Dynamic Interferometry Based on Hertz-Level Low Differential-Frequency Acousto-Optic Frequency Shifter. SPIE Optical Metrology.

[24] Jiao Q., Zhang Y., Wu Z., Kong X., Lü T., Hao Y., Li B., Zhang W. (2025). Vibration-Resistant Full-Field Heterodyne Long-Arm Interferometry. Chin. Opt. Lett..

[25] Zhao S., Liu A., Yang S., Wu Z., Zhang W. (2025). Analysis and Correction of Phase Calculation Errors Introduced by Residual Coherence in Short-Coherence Heterodyne Interferometry. Opt. Express.

[26] Chen S., Xue S., Wang G., Tian Y. (2017). Subaperture Stitching Algorithms: A Comparison. Opt. Commun..

[27] Murphy P.E., Forbes G.W., Fleig J.F., Miladinovic D., DeVries G., O’Donohue S. (2006). Recent Advances in Subaperture Stitching Interferometry. Optical Fabrication and Testing.

[28] Yang J., Meng Y., Hu X., Yang T., Wang Z., Jia D., Ge C. (2024). High-Ranging-Precision FMCW LiDAR with Adaptive Pre-Distortion of Current Injection to a Semiconductor Laser. J. Light. Technol..

[29] Sun C., Shi W., Zhang K., Zhang A. (2026). High-Precision FMCW LiDAR With Simultaneously Acquired and Stitched Multiple Chirps. IEEE Trans. Instrum. Meas..

[30] Sun C., Chen Z., Ye S., Lin J., Shi W., Li B., Teng F., Li X., Zhang A. (2023). Highly-Time-Resolved FMCW LiDAR with Synchronously-Nonlinearity-Corrected Acquisition for Dynamic Locomotion. Opt. Express.

[31] Sun C., Liu W., Li B., Shi W., Lin J., Zhang A. (2023). Efficient Blue-Green Light Phased Array Based on High-Contrast Grating as a Demultiplexer. IEEE Photonics Technol. Lett..

[32] Poulton C.V., Byrd M.J., Russo P., Moss B., Shatrovoy O., Khandaker M., Watts M.R. (2022). Coherent LiDAR With an 8192-Element Optical Phased Array and Driving Laser. IEEE J. Sel. Top. Quantum Electron..

[33] Chen J., Li W., Lian D., Zhao S., Dai D., Shi Y. (2024). Coaxial Transceiving LiDAR Based on a Silicon Photonic Optical Phased Array. Opt. Lett..

[34] Sun C., Chen Z., Teng F., Wang Q., Lin J., Li B., Shi W., Jia D., Li X., Zhang A. (2023). Dynamic Frequency-Modulated Continuous-Wave LiDAR Coupled Through a Rotary Interface. J. Light. Technol..

[35] Wang H., Chen Z., Sun C., Deng S., Tang X., Zhang L., Jiang R., Shi W., Chen Z., Li Z. (2021). Broadband Silicon Nitride Nanophotonic Phased Arrays for Wide-Angle Beam Steering. Opt. Lett..

